# High Virulence of *Wolbachia* after Host Switching: When Autophagy Hurts

**DOI:** 10.1371/journal.ppat.1002844

**Published:** 2012-08-02

**Authors:** Winka Le Clec'h, Christine Braquart-Varnier, Maryline Raimond, Jean-Baptiste Ferdy, Didier Bouchon, Mathieu Sicard

**Affiliations:** 1 Laboratoire Ecologie et Biologie des Interactions, UMR CNRS 7267, Equipe Ecologie Evolution Symbiose, Université de Poitiers, Poitiers, France; 2 Evolution et Diversité Biologique, UMR CNRS 5174, Université Paul Sabatier - Toulouse III, Toulouse, France; Yale School of Public Health, United States of America

## Abstract

*Wolbachia* are widespread endosymbionts found in a large variety of arthropods. While these bacteria are generally transmitted vertically and exhibit weak virulence in their native hosts, a growing number of studies suggests that horizontal transfers of *Wolbachia* to new host species also occur frequently in nature. In transfer situations, virulence variations can be predicted since hosts and symbionts are not adapted to each other. Here, we describe a situation where a *Wolbachia* strain (*w*VulC) becomes a pathogen when transfected from its native terrestrial isopod host species (*Armadillidium vulgare*) to another species (*Porcellio d. dilatatus*). Such transfer of *w*VulC kills all recipient animals within 75 days. Before death, animals suffer symptoms such as growth slowdown and nervous system disorders. Neither those symptoms nor mortalities were observed after injection of *w*VulC into its native host *A. vulgare*. Analyses of *w*VulC's densities in main organs including Central Nervous System (CNS) of both naturally infected *A. vulgare* and transfected *P. d. dilatatus* and *A. vulgare* individuals revealed a similar pattern of host colonization suggesting an overall similar resistance of both host species towards this bacterium. However, for only *P. d. dilatatus*, we observed drastic accumulations of autophagic vesicles and vacuoles in the nerve cells and adipocytes of the CNS from individuals infected by *w*VulC. The symptoms and mortalities could therefore be explained by this huge autophagic response against *w*VulC in *P. d. dilatatus* cells that is not triggered in *A. vulgare*. Our results show that *Wolbachia* (*w*VulC) can lead to a pathogenic interaction when transferred horizontally into species that are phylogenetically close to their native hosts. This change in virulence likely results from the autophagic response of the host, strongly altering its tolerance to the symbiont and turning it into a deadly pathogen.

## Author Summary

Characterizing the causes of a virulence increase when a parasite jumps from one host species to another is fundamental to the understanding of disease emergence. In this context, we studied the bacterium *Wolbachia w*VulC, a natural symbiont of one terrestrial isopod species that becomes a pathogen when transfected into individuals of another species. Before death, recipient animals suffer various symptoms including nervous system disorders caused by the multiplication of *w*VulC. Interestingly, the quantification of *w*VulC loads showed similar titers in the individuals from both the recipient and native species. The difference between the two host species lies in the way they respond to the invasion of *w*VulC and not in their resistance *per se*: While the recipient host species exhibits an acute autophagic response leading to central nervous system cells disorganization, this phenomenon was not observed in the native host species, which seems to better tolerate the bacterium. Together, our results show that tolerance can be a better evolutionary strategy to counteract parasite damage than to activate a putative resistance pathway which, as a double-edged sword, can arm the host itself and increase the virulence of a parasite.

## Introduction

*Wolbachia pipientis* are intracellular α-proteobacteria which are extensively distributed among arthropods and filarial nematodes. These bacteria are vertically transmitted from female hosts to their offspring. The presence of *Wolbachia* induces a number of reproductive perturbations for their hosts including cytoplasmic incompatibility (CI) [1], [2], male killing [3], modification of parthenogenesis [4] and feminization [5], all enhancing the vertical spread of infection in host populations. The *Wolbachia*, in their wide range of hosts, are involved in diverse interactions in terms of costs and benefits to their host that can be placed on a continuum from mutualism to parasitism [6]. However, the global picture is that *Wolbachia* are weakly virulent to their native hosts [7], [8]. This low virulence can be explained by the evolutionary consequences of vertical transmission that theoretically leads to the attenuation of symbiont virulence through a co-evolutionary process with its host [9], [10]. However, the general lack of congruence between *Wolbachia* and host phylogenies suggests that *Wolbachia* frequently colonize new hosts through horizontal transfer [6], [11] and that this mode of spreading may be fundamental to their evolutionary dynamics [12].

Immediately after transfer into a new host species, the virulence of *Wolbachia* is expected to differ from that expressed in its native host because of a lack of host/symbiont co-adaptation, which can only occur after several host generations. Two main non-exclusive situations have been proposed to explain the emergence of a higher level of virulence when a symbiont colonizes a new host: (1) maladapted symbionts can overexploit their host, by multiplying too fast or in a “wrong” compartment (*e.g.* in organs other than gonads, where *Wolbachia* are typically found), (2) maladapted hosts could respond to the presence of an unknown symbiont by employing inefficient and very costly immune defences [13]. In the first situation, the virulence comes primarily from an overexploitation of host resources by the parasites, and the new host can be considered as less resistant because parasite burden is higher in some tissues of the new host than in the native one. In the second situation, the immune system normally dedicated to the defence of organisms becomes a double-edged sword, turning itself against the host. A disproportionately strong response can lead to an immunopathology that can in turn deeply harm the hosts. Therefore, being able to tolerate the multiplication of parasites to a certain extent by not activating some immune pathways can constitute a good strategy to limit virulence [14]. It is thus possible that through the co-evolutionary process taking place between the *Wolbachia* and their hosts, especially as prevalence is often high, hosts quickly select either a way to attempt to control *Wolbachia* proliferation or at the opposite, a way to better tolerate the presence of the symbionts. Being able to decrease invaders load would reduce their cost for the hosts while increasing tolerance would avoid both the direct (*i.e.* production of immune effectors) and indirect costs (*i.e.* the damage caused by immune effectors) of the immune response.

To address the question of the causes of drastic change in virulence of a symbiont, we studied a situation where a *Wolbachia*-host interaction becomes pathogenic when the bacteria (*w*VulC) are transferred from its native terrestrial isopod species host (*Armadillidium vulgare*) to another one (*Porcellio d. dilatatus*) [15], [16]. In its native host *A. vulgare*, *w*VulC not only colonizes gonads for vertical transmission but is also found in somatic tissues [17], [18]. This extensive colonization could play a role in the virulence of *w*VulC detected in several *A. vulgare*'s life history traits: Reduction in hemocyte load, phenoloxydase activity, reproduction and survival [19], [20]. However, though *w*VulC is clearly involved in a conflicting interaction with *A. vulgare* having negative impacts on several of its life history traits, this virulence is weak and *Wolbachia*-infected individuals usually live for years [19]. The aim of the present study was to understand how *Wolbachia w*VulC becomes a pathogen when introduced in the new host *Porcellio d. dilatatus*. To do so, we focused our work on the analysis of the different symptoms that appear prior death of the recipient host *Porcellio d. dilatatus*. For this, several host life history traits have been measured (gain of weight, mobility, burying behaviour). In parallel, we assessed *Wolbachia w*VulC multiplication in gonads, hemocytes (*i.e* immune cells) and CNS (*i.e.* nerve cells and neighbouring adipocytes). Moreover, we observed gonads and CNS cells by electron microscopy. We demonstrate that infection with the strain *w*VulC causes acute disease to *P. d. dilatatus* with symptoms of paralysis that indicate the existence of nervous system disorders. We suggest that these symptoms and the death that follows are linked to the autophagic reaction observed in the CNS cells of *P. d. dilatatus* but not in *A. vulgare* which seems thus more tolerant to its native *Wolbachia* strain it co-evolved with.

## Results

### Quantification of *w*VulC and *w*Dil in inoculum

In order to quantify the dose of *Wolbachia* injected into recipient hosts that could vary due to the donor host species (*A. vulgare* for *w*VulC and *P. d. dilatatus* for *w*Dil), we performed quantification by qPCR on the *wsp* gene. We found that the quantities of *Wolbachia* cells inoculated in recipient hosts significantly differed between treatments (*i.e. w*VulC *versus w*Dil). This is because the natural amount of *Wolbachia* present in the ovaries of *A. vulgare*, the donor host species of *w*VulC (mean dose ± se = 1.66×10^5^±6.30×10^4^ *wsp* copies/injected µL) is ten times higher than in *Porcellio d. dilatatus*, the donor host species of *w*Dil (mean dose ± se = 1.14×10^4^±3.60×10^3^ *wsp* copies/injected µL); (comparison by t-test = 2.9956 *df* = 4 *p* = 0.0401). To evaluate the influence of the injected dose in the effect observed on the recipient hosts, we tested the effect of *w*VulC with a very small inoculum (3.02×10^3^±2.60×10^3^ *wsp* copies/injected µL) by diluting the initial inoculum a hundred times. Moreover, to take into account the differences between injected doses in a statistical framework, the effect of the factor “dose” was tested at first to absorb as much variance as possible, and then it was therefore possible to test the effect of each strain of *Wolbachia per se* independently from the difference in injected dose.

### *wsp* gene sequencing from inoculum and transfected animals

To verify that only the expected *Wolbachia* strain was present (*i.e. w*Dil or *w*VulC) in the inoculum and in transfected *A. vulgare* and *P. d. dilatatus*, we amplified and sequenced 610 bp of the *Wolbachia* surface protein gene (*wsp*) in (i) all inoculum and (ii) recipient animals at 60 days Post Injection (PI). In all the cases, the chromatograms obtained showed well resolved peaks indicating that only one matrix has been sequenced per host or inoculum. The comparison of all the obtained sequences with the reference *wsp* sequences of *w*Dil and *w*VulC showed no variation. In all cases we therefore found the strain of *Wolbachia* which was expected in the given sample.

### No significant effect of *Wolbachia* injection on *Armadillidium vulgare*'s life history traits

**Figure 1:**
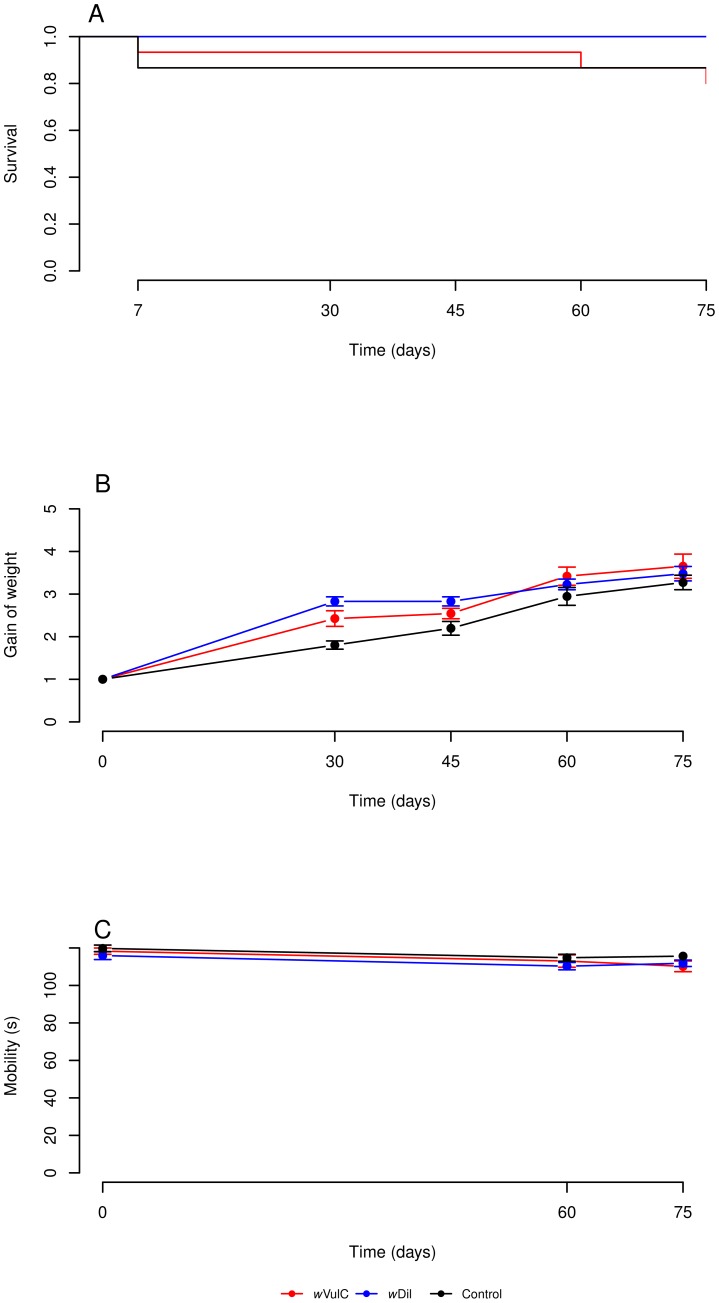
Survival, gain of weight and mobility plots for *Armadillidium vulgare* during 75 days post-injection. There were no significant differences between animals injected with *w*VulC, *w*Dil or control treatment for all these life history traits: Survival (A), Gain of weight (B) and Mobility (C).

After the injections of *w*VulC or *w*Dil, no significant effects were detected for *A. vulgare* females on all the different life history traits measured (gain of weight, activity and survival) (Table 1 for statistics; Figure 1). Moreover, no nervous disorders symptoms such as seizures and tremors were observed for *A. vulgare*.

**Table 1:** Statistical analyses ran on the life history traits data obtained for female *A. vulgare* injected by *w*VulC, *w*Dil and control treatment.

		Global comparison (control/*w*VulC/*w*Dil)			Comparison between effect of *w*VulC/*w*Dil		
	Factor	*df*	deviance	*p*	*df*	deviance	*p*
**Gain of weight**	**Dose**	1	3.5268	0.0716	1	3.1814	0.0720
(lme analyse)	**Treatment**	2	3.9370	0.1396	1	3.1303	0.0768
	**Residuals**	12	0.0210	#	8	0.0210	#
**Mobility**	**Dose**	1	3.2843	0.0699	1	2.6187	0.1056
(lme analyse)	**Treatment**	2	4.5969	0.1004	1	1.6130	0.2040
	**Residuals**	12	8.2608	#	8	8.2608	#
**Survival**	**Dose**	1	1.1698	0.2794	1	1.1946	0.2743
(Cox model analyse)	**Treatment**	2	5.1525	0.0760	1	3.1256	0.0712
	**Residuals**	12	0.0006	#	8	0.0547	#

The gain of weight and mobility were compared between treatments at 60 days post-injection with a Gaussian linear mixed-effects model with randomized block effect fitted by maximum likelihood (ML). Survival curves obtained with the different treatments were compared using a general mixed-effects Cox model with randomized block effect.

### Perturbation of *Porcellio d. dilatatus*'s life history traits by *w*VulC

#### Gain of weight

**Figure 2:**
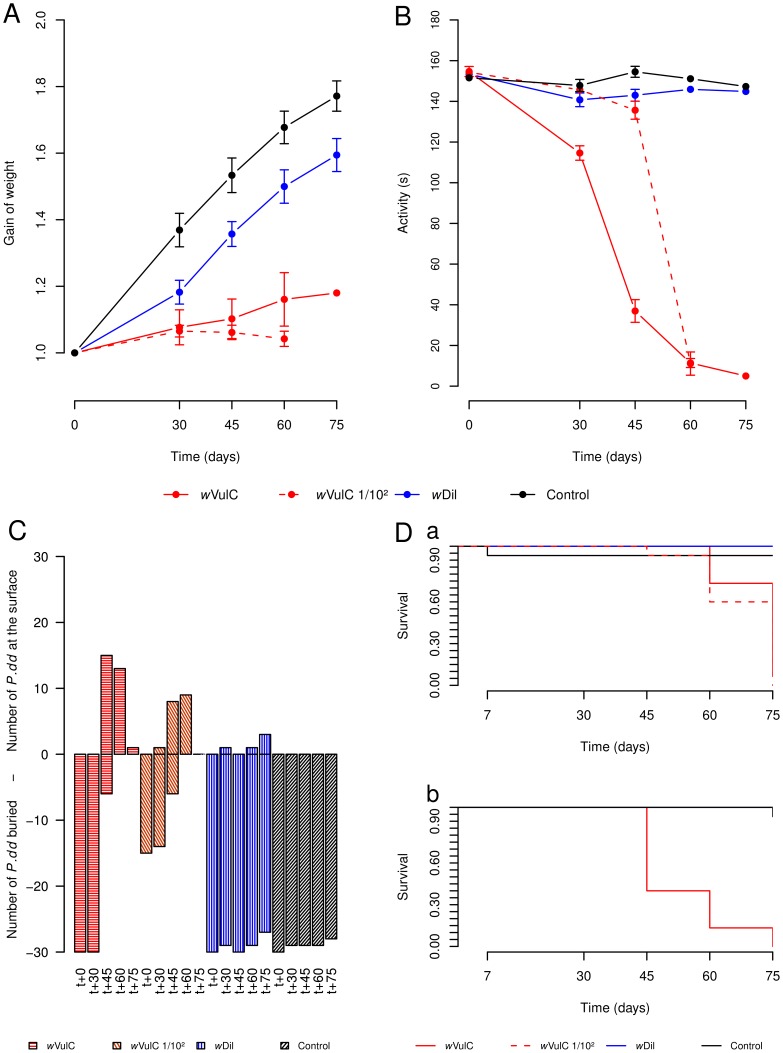
Gain of weight, mobility, burying, survival plots for *Porcellio dilatatus*. Animals were injected by pure suspension of *w*VulC, diluted *w*VulC inoculum (only for females), *w*Dil or control treatment and monitored during 75 days post-injection. (A) Gain of weight: Both *Wolbachia* strains negatively impact this parameter compared to the control treatment. However, *w*VulC (*i.e* pure or 1/100 diluted inoculum) induces a lower growth rate than the native strain *w*Dil. (B) Mobility: this parameter is dramatically reduced for animals injected by *w*VulC compared to those from other treatments. On the opposite, injection of *w*Dil has no significant effect on the host's mobility. (C) Burying behaviour: The level 0 corresponds to the surface of the substrate, negative values correspond to the number of buried animals while positive values correspond to the number of animals found at the surface of the substrate. For control treatment animals, not a single individual rose up at the surface and for individuals injected by *w*Dil only few animals were spotted at the surface. When inoculated by *w*VulC, all animals rose up to the substrate after 60 days. (D) Survival (a) data for females and (b) data for males: The injection of *w*VulC kills all the individuals. However, the males die quicker than the females. No extra-mortalities were reported for injection with *w*Dil compared to control treatment.

A global analysis showed that 60 days post-injection, most of the variance in gain of weight between samples was explained by differences among treatments (*i.e.* control, injection of *w*VulC and injection of *w*Dil) but not by difference between sex or the injected dose of *Wolbachia* (Table 2). The same model ran after removing the control treatment from the dataset, demonstrates that variations in gain of weight depends on the *Wolbachia* strain (*w*VulC *versus w*Dil): *w*VulC induced a stronger growth slowdown than *w*Dil (respective means for gain of weight ± se = 1.1000±0.08 and 1.4900±0.05 after 60 days following injection; Figure 2A; Table 2). The animals injected with the 1/100 diluted *w*VulC inoculum suffered a similar growth slowdown compared to those injected with the pure suspension of *w*VulC (ANOVA, F = 0.1384 *df* = 1,15 *p* = 0.7150; Figure 2A).

**Table 2:** Statistical analyses ran on the life history traits data obtained for *P. d. dilatatus* injected by *w*VulC (pure and diluted inoculum), *w*Dil and control treatment.

		Global comparison (control/*w*VulC/*w*Dil)			Comparison between effect of *w*VulC/*w*Dil		
	Factor	*df*	deviance	*p*	*df*	deviance	*p*
**Gain of weight**	**Sex**	1	0.0048	0.9447	1	0.0880	0.7946
(lme analyse)	**Dose**	1	1.3670	0.2423	1	0.5347	0.4646
	**Treatment**	**2**	**6.5895**	**0.0370**	**1**	**9.6610**	**0.0018**
	**Residuals**	26	0.0103	#	17	0.0109	#
**Mobility**	**Sex**	1	0.0412	0.8391	1	0.0614	0.8042
(lme analyse)	**Dose**	1	0.2593	0.6105	1	0.1633	0.6861
	**Treatment**	**2**	**76.6916**	**<0.0001**	**1**	**51.2569**	**<0.0001**
	**Residuals**	26	7.2512	#	10	7.5134	#
**Surface location**	**Sex**	1	0.0160	0.9004	1	0.0160	0.9004
(glm analyse)	**Dose**	1	0.8160	0.3662	1	0.8160	0.3662
	**Treatment**	**2**	**173.141**	**<0.0001**	**1**	**118.812**	**<0.0001**
	**Residuals**	86	19.3036	#	58	19.3036	#
**Survival**	**Sex**	**1**	**8.1314**	**0.0043**	**1**	**9.1004**	**0.0025**
(Cox Model analyse)	**Dose**	1	0.1081	0.7422	1	0.1901	0.6627
	**Treatment**	**2**	**39.8903**	**<0.0001**	**1**	**37.2365**	**<0.0001**
	**Residuals**	26	0.0083	#	17	0.0083	#

The gain of weight and mobility were compared between treatments at 60 days post-injection with a Gaussian linear mixed-effects model with randomized block effect fitted by maximum likelihood (ML). For comparison of the *P. d. dilatatus* location in boxes between treatments through the whole experiment, a generalized linear model with binomial error and logit link function was performed. Survival curves were compared using a general mixed-effects Cox model with randomized block effect as for *A. vulgare*.

#### Mobility

A global analysis showed that 60 days post-injection, the variance in mobility between animals was due mostly to differences among treatments (*i.e.* injection of *w*VulC or *w*Dil or control) and did not relate to sex or the amount of *Wolbachia* cells injected (Table 2). A strong differential effect on mobility was induced according to the injected strain (*w*VulC or *w*Dil). Between 30 and 75 days, the mobility of individuals injected by *w*VulC observed during 180 s strongly dropped from 115±3.55 s to 0 s while animals injected with the native strain *w*Dil or the control treatment did not exhibit reduction in mobility (150±2.45 s.; Figure 2B; Video S1). Moreover, starting from 45 days post-injection, *P. d. dilatatus* individuals injected with *w*VulC turned to asthenia (*i.e.* they seemed “lax”), suffering from seizures and leg tremors. These symptoms were observed after injection of both pure suspension of *w*VulC and 1/100 diluted suspension of *w*VulC (Figure 2B; Video S1).

#### Buried or not?

Another change in the behaviour of *P. d. dilatatus* observed after *Wolbachia* injection was the location in the rearing box substrate. The normal behaviour of aposymbiotic *P. d. dilatatus* is to bury themselves into the substrate. But after *Wolbachia*'s injections, some males and females rose at the surface (Figure 2C; Video S1). The analysis of data with a binomial general linear model showed that the factor that statistically explained most of the variability in location was again the type of *Wolbachia* strain injected (Table 2). Between 45 and 60 days post-injection, most of the individuals injected with the pure or 1/100 diluted inoculum of *w*VulC strain were found at the surface (respectively 100% and 57 to 72% of the animals) while after *w*Dil injection, only 6.7% of animals were spotted at the surface over the whole 75 days observation period. For the control treatment the situation was totally opposite to the one observed with *w*VulC since not a single animal was found at the surface of the substrate over the 75 days period of observation (Figure 2C; Video S1).

#### Survival

The above described symptoms for the *P. d. dilatatus* injected with the pure and diluted suspensions of *w*VulC (*i.e.* decrease in gain of weight, mobility and number of animals buried) were followed by death of all the animals (Figure 2D). Comparison of survival plots between groups of individuals of *P. d. dilatatus* injected with *w*VulC, *w*Dil and control treatments using general mixed-effects Cox model revealed significant differences. While in control and *w*Dil treatments, only 2 mortality cases were observed during the whole experiment, all 30 individuals injected with *w*VulC died within 75 days (Table 2; Figure 2D). Moreover, clear differences in survival curves were reported between males and females injected with *w*VulC (Table 2; Figure 2D). The males infected with *w*VulC died quicker than the females (the TL_50_ was respectively 45 days and 75 days). Since to reproduce *P. d. dilatatus* needs at least 50 days from insemination to the releasing of the young from the marsupium no vertical transmission (*i.e* from transinfected mother to the progeny) could be possible after the injection of the pathogenic *Wolbachia* strain *w*VulC. As the pathologic effects rose within the first month post injection and that the bacteria would need at least a month to well colonize the ovaries vertical transmission of *w*VulC in *P. d. dilatatus* is clearly not possible.

### *Wolbachia* multiplication in CNS, gonads and hemocytes of *P. d. dilatatus* and *A. vulgare*

**Figure 3:**
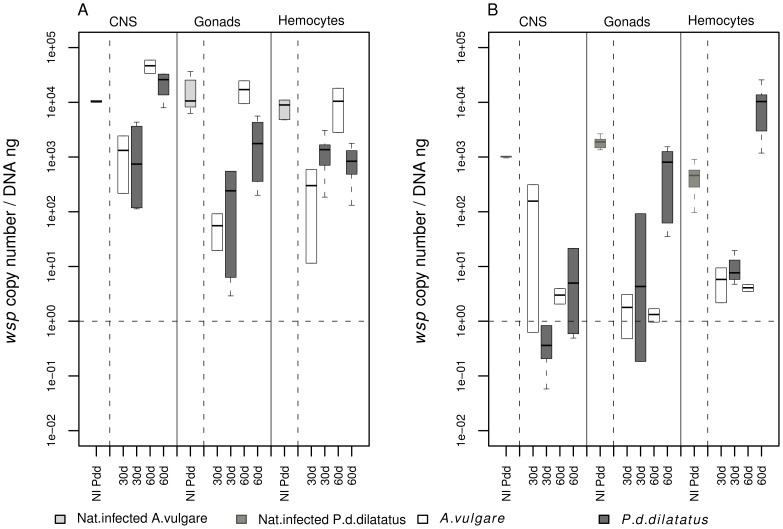
*Wolbachia* loads in CNS, gonads and hemocytes of *Porcellio d. dilatatus* and *Armadillidium vulgare*. (A) Comparison between (i) *P. d. dilatatus* in dark grey and *A. vulgare* in white injected with *w*VulC and (ii) naturally infected *A. vulgare* in light grey revealed no significant differences for the *Wolbachia* loads in CNS cells, gonads and hemocytes (*i.e* immune cells), at both 30 and 60 days post-injection. (B) Comparison between (i) *P. d. dilatatus* in dark grey and *A. vulgare* in white injected with *w*Dil and (ii) naturally infected *P. d. dilatatus* in dim grey revealed no significant differences for the *Wolbachia* loads in CNS cells at both 30 and 60 days PI. However, at 60 days post-injection, for gonads and hemocytes *w*Dil loads were significantly higher in the native host *P. d. dilatatus* than in *A. vulgare*. A global comparison between *w*Dil and *w*VulC loads showed that *w*VulC (A) always exhibited higher bacterial loads than *w*Dil (B) in both host species.

In order to assess the global replication dynamics of *Wolbachia* in their hosts, *Wolbachia* quantifications were performed in CNS, gonads and hemocytes (*i.e.* immune cells). These quantifications were performed at 30 days and 60 days post injection for both recipient host species. The bacterial load of *w*VulC in CNS, which is composed of a core of nerve cells surrounded by a sheathing of adipocytes but also in gonads and hemocytes did not significantly differ between the two host species (in CNS at 30 days PI t-test = −1.1901 *df* = 1 *p* = 0.4449 and at 60 days PI t-test = 1.048 *df* = 1 *p* = 0.4851; in gonads at 30 days t-test = −1.1758 *df* = 1 *p* = 0.4489 and at 60 days t-test = 1.4303 *df* = 1 *p* = 0.3884; in hemocytes at 30 days t-test = −0.5327 *df* = 1 *p* = 0.6884 and at 60 days t-test = 1.1216 *df* = 1 *p* = 0.4635; Figure 3A). Moreover, the bacterial loads at 60 days post-injection were similar to those obtained in the CNS, gonads and hemocytes of a naturally infected *A. vulgare* (*i.e.* which received *w*VulC vertically from their mothers) (in CNS t-test = −2.1411 *df* = 3 *p* = 0.1217, in gonads t-test = 2.635 *df* = 3 *p* = 0.0779 and in hemocytes t-test = 1.6100 *df* = 3 *p* = 0.2487; Figure 3A). For individuals of both recipient host species injected with *w*Dil, at both 30 days and 60 days post-injections, we showed no significant difference between bacterial load in CNS (at 30 days t-test = 1.0107 *df* = 1 *p* = 0.4966; at 60 days t-test = 1.088 *df* = 1 *p* = 0.4732; Figure 3B). In the other tested tissues (*i.e* gonads and hemocytes), we obtained significant differences when comparing the *w*Dil quantification at 60 days post-injection between transfected *P. d. dilatatus* and *A. vulgare* (in gonads t-test = 2.9053 *df* = 5 *p* = 0.0335 and in hemocytes t-test = −2.9867 *df* = 5 *p* = 0.0305; Figure 3B). In these two cases, *w*Dil densities were higher in the native host *P. d. dilatatus* than in *A. vulgare* (Figure 3B).

The quantifications also showed that the density of *w*Dil in CNS of all injected animals from both host species or in naturally infected *P. d. dilatatus* was always ten to a hundred times lower than the density of *w*VulC injected in the same animals or in naturally infected *A. vulgare* (t-test = −37.0596 *df* = 4 *p*<0.0001).

### Autophagic vesicles accumulation in nervous cells of *P. d. dilatatus* infected by *w*VulC

**Figure 4:**
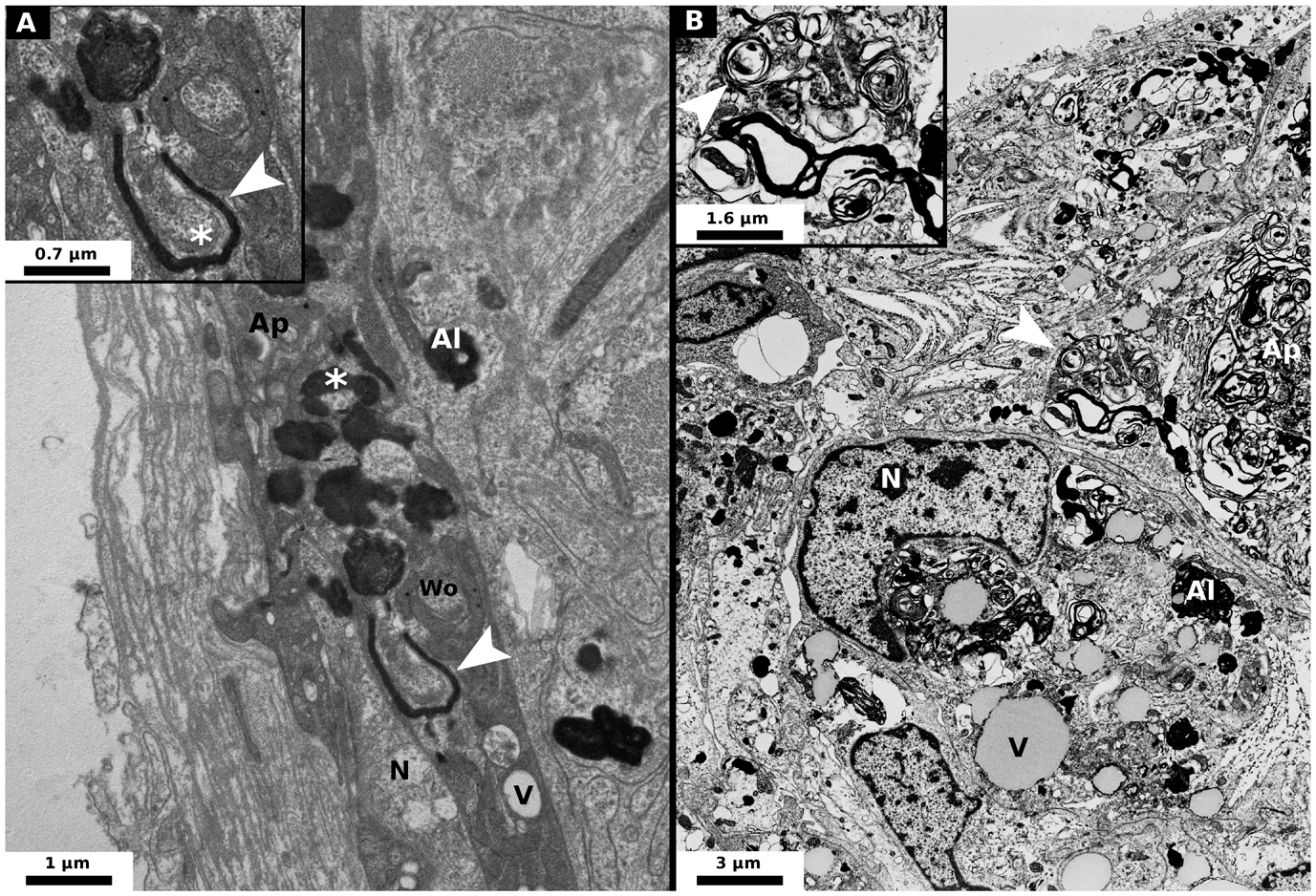
Micrograph of nerve cells of *Porcellio d. dilatatus* injected by *w*VulC. These cells are localised in the CNS ganglion. (A) At 30 days post-injection, some autolysosomes and bacteria are visible: autophagic process is already quite high compared to control [N: Nucleus, V: Vacuole, Ap: Autophagosomes (Autophagic hallmark), Al: Autolysosomes, Wo: *Wolbachia*, *: a *Wolbachia* which seems to be surrounded by an autolysosome, Arrow head: magnified area]. (B) At 60 days post-injection, the nerve cells are severely damaged by the accumulation of autophagic vesicles (N: Nucleus, V: Vacuole, Ap: Autophagosomes (Autophagic hallmark), Al: Autolysosomes, Arrow head: magnified area and probably a *Wolbachia* in autophagic degradation).

**Figure 5:**
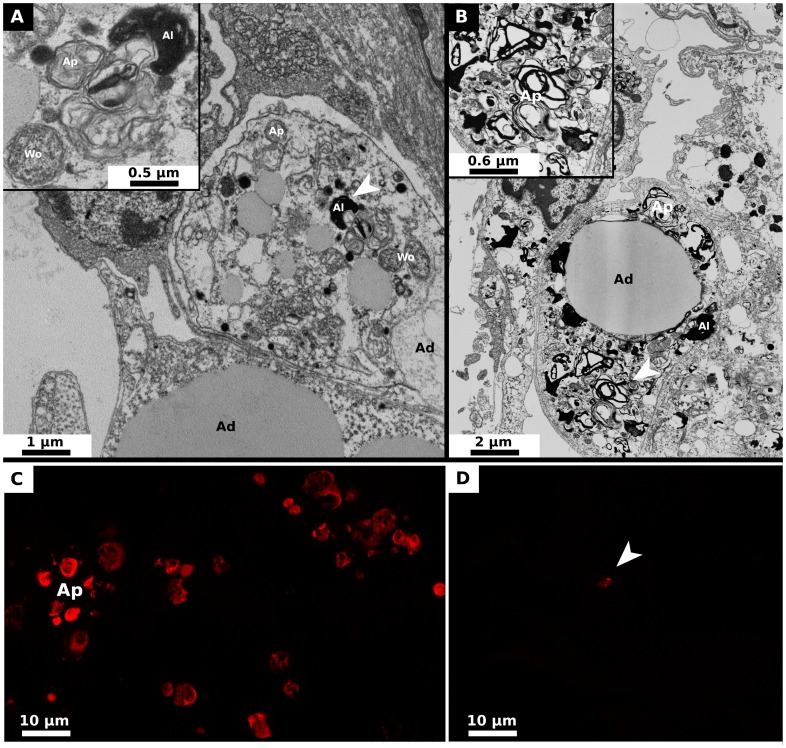
Micrograph and LC3B antibody labelling of adipocytes neighboring the nerve cells of *Porcellio d. dilatatus*. These adipocytes are localised around the nerve cells of the CNS and constitutes the sheathing of this organ. (A) At 30 days post-injection, autolysosomes, autophagosomes but also bacteria were observed: autophagic process was already higher compared to the control (Ad: adipocyte, Ap: Autophagosomes (Autophagic hallmark), Al: Autolysosomes, Wo: *Wolbachia*, Arrow head: magnified area). (B) At 60 days post-injection, the adipocytes were severely damaged by the accumulation of autophagic vesicles (Ad: adipocyte, Ap: Autophagosomes, Al: Autolysosomes, Wo: *Wolbachia*, Arrow head: magnified area). (C) With the antibody labelling anti-LC3B, the protein LC3B implicated in autophagic processes was detected in adipocytes of *P. d. dilatatus* injected with wVulC at 45 days post-injection. The highly labeled spherical structures would reflect the incorporation of the LC3B protein in the phagophores. (D) With adipocytes of *P. d. dilatatus* injected by *w*Dil but also in other tested situations, only really few of these structures were detected by epiflorescent microscopy.

For *A. vulgare* individuals injected with either *w*Dil or *w*VulC but also for those which were naturally infected with *w*VulC, nerve cells from the CNS and adipocytes but also gonads observed by transmission electron microscopy at both 30 and 60 days post-injections were normal (*i.e.* nucleus and organelles exhibited normal shapes, no autophagic phagosomes and only few lysosomes were present in the cytoplasm). The same cell types observed in *P. d. dilatatus* injected with its native *Wolbachia* strain *w*Dil at both 30 and 60 days post-injections were also normal. By contrast, strong alterations of the structure of the cells were observed only in the CNS and adipocytes but not in gonads of *P. d. dilatatus* injected with *w*VulC. At 30 days post injection, in both adipocytes and nerve cells, we observed some *Wolbachia* cells and high densities of autophagosomes and autolysosomes which are characteristic of autophagic process (Figure 4A and 5A). Moreover, in one nerve cell, we specifically observed the formation of an autophagosome around a *Wolbachia* cell (Figure 4A). At 60 days post injection, the autophagic process observed in the nerve cells and adipocytes clearly amplified compared to what was observed at 30 days post-injections. The CNS cells were severely damaged. Both nerve cells and adipocytes were filled with autophagic vesicles and the organelles were less visible because of a complete disorganization of the cytoplasm (Figure 4B and 5B). To have an additional confirmation of the occurrence of autophagy, we applied the antibody labelling kit LC3B (Invitrogen) on adipocytes of *A. vulgare* and *P. d. dilatatus* injected with *w*VulC or *w*Dil. This approach confirmed a high autophagic activity in the adipocytes collected in *P. d. dilatatus* individuals that were injected with *w*VulC (Figure 5C). For such individuals, we observed highly labeled spherical structures that would reflect the incorporation of the LC3B protein in the phagophores. This kind of phagophore was very rarely observed for *A. vulgare* and for other treatments in *P. d. dilatatus* (Figure 5D).

## Discussion

While *Wolbachia* are typically transmitted vertically within host populations, it is increasingly recognized that horizontal transfer between host species plays an important role in the evolutionary dynamics of these endosymbionts in arthropods [11]. In this study, we aimed at assessing the potential change in virulence of a *Wolbachia* strain (*w*VulC) immediately after horizontal transfer into a new host, as well as characterizing the causes of this virulence change. We show that after experimental transfer of *w*VulC from its native terrestrial isopod host species (*A. vulgare*) to a closely related one (*P. d. dilatatus*), the virulence increases so much that it becomes a pathogen. Such a high virulence is remarkable and is reminiscent of the observations made for another *Wolbachia* strain, *w*MelPop which causes early death of both its native and foreign hosts [21]–[23]. In the case of *w*MelPop, this high virulence has been proposed to be caused by an abnormally high bacterial load in host cells, especially in nerve ones [21]. Along with high loads of *w*Melpop in nerve cells, important behavioural modifications have been recorded after horizontal transfer followed by vertical transmission in mosquitoes, suggesting an alteration of the central nervous system (CNS) [24], [25]. In the present study, the characterization of symptoms such as paralysis, seizures and leg tremors observed prior to the death of *P. d. dilatatus* injected by *w*VulC, led us to hypothesize that the sickness induced by this *Wolbachia* strain could also involve nervous system disorders.

The quantifications of the *Wolbachia* in the main organs of both *A. vulgare* and *P. d. dilatatus* individuals that received the bacteria either vertically from their mothers or horizontally by experimental injection strengthened this hypothesis. We found that *w*VulC which is virulent in *P. d. dilatatus* exhibited bacterial loads in the CNS that were at least 10 times higher than those reached by the avirulent *w*Dil. Therefore, similarly to what was observed for *w*Melpop, the high bacterial loads reached by *w*VulC in the CNS certainly constitutes one component of its high virulence. The multiplication of intracellular bacteria in nerve cells as a cause of CNS diseases has also been extensively described in vertebrates [26]. Interestingly, most of the intracellular bacteria that cause such diseases belong to the group of Rickettsiales, to which *Wolbachia* also belongs [26], [27]. Some of these Rickettsiales, such as *Rickettsia sp.* have even been shown to be responsible for diseases like encephalitis and meningitidis in humans [26]. It is also noteworthy that symptoms similar to those caused by *w*VulC and *w*Melpop in arthropods such as reduction of movements, uncontrolled tremors and inability to feed properly have been described in important CNS diseases caused by Rickettsiales in other animals [24], [26], [28].

Nevertheless high bacterial loads in CNS but also in gonads and hemocytes are unlikely to fully explain the pathogenesis caused by *w*VulC in *P. d. dilatatus* because *A. vulgare* individuals infected vertically or horizontally with this strain survive similar infection pattern [19]. Indeed, *w*VulC reaches similar bacterial loads in the main organs of both its native and foreign host suggesting that both isopod species have an overall similar resistance [14]. We therefore suspected that the pathogenicity of this strain towards *P. d. dilatatus* could be mostly due to a lower ability of this foreign host to tolerate the multiplication of *w*VulC compared to *A. vulgare* [14], [29]. One hypothesis would be that *A. vulgare*, through vertical co-evolution with *w*VulC, has increased its ability to tolerate the high bacterial loads in its CNS. By contrast, *P. d. dilatatus* could not evolve towards tolerating high bacterial loads in its CNS because it vertically co-evolved with a strain (*w*Dil) that exhibits much lower bacterial loads in this organ than *w*VulC.

The main factor explaining the different virulence of *w*VulC observed between the two isopod species would thus be the way infected tissues respond to the multiplication of *w*VulC. In agreement with this hypothesis, electron microscopy and LC3B labelling revealed marked differences in the autophagic response of the host cells between *A. vulgare* and *P. d. dilatatus*. While in the former species both adipocytes and nerve cells did not show any sign of deregulated autophagy, in *P. d. dilatatus*, we observed that these two different cell types were completely filled with autophagic vesicles, resulting in a profound disorganization of their cytoplasm. These observations suggest that in addition to high bacterial loads in the CNS cells, the disproportionate autophagic response of these cells is likely to be a major component of the CNS disease caused by *w*VulC in *P. d. dilatatus*.

Autophagy even if really conserved among eukaryotes has only quite recently been considered as a way to regulate intracellular parasites [30]. A role for autophagy in the response to *Wolbachia* infection has not previously been clearly described, but a recent paper described the presence of autophagosomes putatively involved in the degradation of dying *w*Melpop in ovaries of *D. melanogaster* [31]. Moreover, *Anaplasma phagocytophilum*, a pathogen closely related to *Wolbachia*, has been shown to manipulate its host's autophagic machinery [32]. Given the importance of autophagy as a multi-pronged defense against intracellular microbes, we propose that the pathogenic effect of *w*VulC onto *P. d. dilatatus* could be an immunopathology characterized by *Wolbachia*-induced disturbance of host autophagic processes, as already described for other intracellular pathogens [33], [34]. The mechanism that causes the activation of an apparently unregulated autophagic response in this novel host is not known. However, for *Anaplasma phagocytophilum*, a previous study has unveiled the importance of the type IV secretion system (T4SS) in the induction and subversion of autophagy [35], [36]. In the genome of *w*VulC strain, genes corresponding to the minimum components for a typical and functional type IV secretion system (T4SS) were identified [37], [38]. This suggests that *w*VulC factors that trigger autophagy may use the T4SS system as an effector translocator to reach their targets. We can thus hypothesize that the strong autophagic response in *P. d. dilatatus* that causes nerve cells destruction could be due to reaction of the host towards the abnormally intense multiplication of *w*VulC in its CNS but also to the production of particular toxins possibly shuttled *via* T4SS. Moreover, it cannot be excluded that the expression of some *w*VulC effectors could change when the bacteria are transfected from native to new host and that this differential expression could play a role in the difference observed in host reactions.

In this study, we show that the *Wolbachia* strain *w*VulC becomes highly virulent when it jumps from its native host (*A. vulgare*) to a new host (*P. d. dilatatus*). This increase in virulence is linked to an apparently unregulated autophagic reaction in the new host which is not observed in the same conditions for the native one. These results suggest that *A. vulgare*, which vertically co-evolved with *w*VulC better tolerates the presence of this bacterium in its cells. In conclusion, our experiments uphold the hypothesis according to which tolerance could be a better evolutionary strategy to counteract parasite damage than activating a putative resistance pathway such as autophagy, which as a double-edged sword can hurt the host and increases the virulence of the parasites.

## Materials and Methods

### Ethic statement

All experimental procedures and animal manipulations did not require an ethics statement.

### Biological materials

All the animals used in this experiment were grown at 20°C in plastic breeding boxes, in natural photoperiod, on moistened potting mix derived from peat from sphagnum moss (pH = 6.4 and conductivity = 50.0 mS/m) with dead lime-tree leaves as a food source. In such laboratory conditions, animals can normally live up to 3 years, whether they are infected or not by *Wolbachia*. The recipient hosts of all experiments were aposymbiotic (*i.e.* without any *Wolbachia*) *Armadillidium vulgare* (originating from the city of Helsingor in Denmark and reared in the laboratory since 1991) and *Porcellio dilatatus dilatatus* which were collecting at the village of Rom in Deux-Sèvres (France) in 1988 and reared since then in the laboratory. As donors of the native strain *w*Dil, we used another lineage of *P. d. dilatatus* naturally infected by *Wolbachia*. These animals were originated from the Sainte-Marguerite Island in Alpes-Maritimes (France) and reared in the laboratory since 2007. As donors of the *Wolbachia* strain *w*VulC, symbiotic *Armadillidium vulgare* originating from the city of Helsingor (Denmark) and reared in the laboratory since 1991 were used.

### *Wolbachia* injection experiments

Aposymbiotic *A. vulgare* (females of 6 months-old) and *P. d. dilatatus* (females and males of 6 months-old) were infected by *w*VulC, *w*Dil or by a control treatment. For each batch of injection, ovary suspensions were prepared with the ovaries of (i) 5 *A. vulgare* symbiotically associated with *w*VulC; (ii) 5 *P. d. dilatatus* symbiotically associated with *w*Dil or (iii) 5 *A. vulgare* that did not host *Wolbachia* for control treatment. The ovaries were collected and crushed into 1 ml of Ringer solution. The resulting suspension was filtered through a 1.2 µm pore membrane, and 1 µL of each filtrate was injected in a small hole pierced in each individual cuticle, using a thin glass needle, into the general cavity, at the posterior part of animals [39]. This protocol was applied to inject 3 independent batches of individuals per treatments (*i.e.* injection of *w*VulC, *w*Dil or control). Animals were then used for life history traits measurements, *Wolbachia* quantifications, electron microscopy and LC3B antibody labeling.

### Animals injected for life history trait measurements

Thirty individuals *per* treatment for *P. d. dilatatus* (*i.e.* 15 males and 15 females) and 15 females *per* treatment for *A. vulgare* were monitored for life history measurements. To be able to demonstrate that the effect of *w*VulC was not due to higher doses injected compared to *w*Dil, 15 *P. d. dilatatus* females were injected with a *w*VulC suspension diluted 100 times with filtered sterile Ringer. For all these animals, life-history traits were recorded during 75 days after the injection.

### Animals injected for *Wolbachia* quantifications

Twelve individuals *per* treatment for *P. d. dilatatus* (*i.e.* 6 males and 6 females) and 6 females *per* treatment for *A. vulgare* were injected for *Wolbachia* quantifications. Half of the animals were used for *Wolbachia* quantification at 30 days post-injection and the other half for *Wolbachia* quantification at 60 days post injection.

### Animals used for electron microscopy and LC3B labeling

For each host species 18 individuals *per* treatment were injected to observe the adipocytes and the nerve cells but also oocytes by electron microscopy at 30 (6 individuals for each treatment) and 60 days (6 individuals for each treatment) post-injection and to perform LC3B labeling antibody kit to characterize autophagic vesicles in adipocytes at 45 days post injections (6 individuals for each treatment).

### Measurements of the different life history traits

For each condition and host species the life history traits were recorded for 75 days post-injection (until the last individuals infected by *w*VulC died for *P. d. dilatatus*). The first measurement was performed at t = 0 then 30 days after the injection. The following measurements were then performed every 15 days until the final one after 75 days. The gain of weight was calculated for each animal at each point by weighting them on a precision balance (d = 0.001 g) and dividing the weight at the time t = x by the weight at time t = 0. The mobility test was performed by measuring the time during which an individual moves in a glass Petri dish onto a period of 180 s. For *P. d. dilatatus*, only we determined the number of individuals located at the surface versus the number of buried animals after opening the breeding box by counting the number of animals found at the substrate and those which were buried. The survival rate was measured by counting the number of live animals for each treatment.

### Tissue samples and DNA extraction for *Wolbachia* quantification

Total DNA was extracted from the Central Nervous system (CNS) (*i.e.* nerve cells and neighbouring adipocytes), the gonads and the hemocytes of each individual as described by Kocher *et al.*, [40] after dissections. For each sample, the concentration and quality (ratios OD 260/280 nm and 260/230 nm) of the extracted DNA were measured using the Nanodrop 1000 spectrophotometer (Thermo).

### Quantification of *Wolbachia* in host's tissues

The quantification of *Wolbachia* by quantitative PCR (qPCR) was performed at 30 and 60 days post-injection to follow the time course of the infections before the death of all *P. d. dilatatus* infected by *w*VulC but after apparition of the first symptoms. All the qPCR amplifications were performed with DNA sampled from the CNS (*i.e.* nerve chord = nerve cells and neighbouring adipocytes), the gonads and the hemocytes. To allow a comparison between animals injected by *Wolbachia* and those which received the bacteria vertically from their mother; we also performed qPCR quantification on respectively 5 females *A. vulgare* naturally infected with *w*VulC and 5 females *P. d. dilatatus* naturally infected with *w*Dil. All the qPCR reactions were performed using Roche LIGHTCYCLER 480 under the following conditions in 10 µL: 5 µL of SYBRGreen MasterMix (Roche), 0.5 µL of 10 µM specific primers *wsp*208f (5′- TGG-TGC-AGC-ATT-TAC-TCC-AG-3′) and *wsp*413r (5′-TCG-CTT-GAT-AAG-CAA-AAC-CA-3′), which amplified 205 bp of a single-copy of the gene *wsp*, 3 µL of sterile water and 1 µL of DNA (between 10 ng and 80 ng of DNA). The thermal cycling used an initial denaturation period of 10 min at 95°C, followed by 45 cycles of denaturing temperature at 95°C for 10 s, the annealing temperature for the reaction was 60°C for 10 s and 72°C for 20 s. A melting curve (65°C to 97°C) was recorded at the end of each reaction in order to check that the PCR product was unique. Efficiency of the PCR reaction was calculated. Standard curve was plotted using 7 dilutions of *wsp* purified PCR product (*wsp* copies.µL-1: 2.63×10^0^, 2.63×10^1^, 2.63×10^2^, 2.63×10^3^, 2.63×10^4^, 2.63×10^5^, 2.63×10^6^, 2.63×10^7^). *wsp* copy number was estimated by calculation in reference to the standard curve. The total DNA quantity (*i.e*. host+*Wolbachia*) of each sample was used to normalize *wsp* gene copy number. The results are thus given in number of *wsp* copies by ng of total DNA. For each condition (individual*organ*Wolbachia strain), two independent technical replicates were performed.

### *wsp* gene amplification and sequencing for *w*VulC strain

In the inoculum and in animals injected with *Wolbachia* 60 days post-injection, we verified that only the expected *Wolbachia* strain was present (*i.e. w*Dil or *w*VulC). To do so, a 610 bp fragment of the *wsp* region of the *Wolbachia* DNA was amplified using the specific primers *wsp*81F and *wsp*691R (81F 5′-TGG TCC AAT AAG TGA TGA AGA AAC and 691R 5′- AAA AAT TAA ACG CTA CTC CA) [41]. The PCR cycling conditions were 95°C for 2 min followed by 35 cycles (95°C for 1 min, 55°C for 1 min, 72°C for 1 min) and 72°C for 5 min. Double strand PCR products were purified using 2 µl of a mix containing two enzymes: 0.05 µl of Antarctic Phosphatase (AnP, 1 u/µl; New England Biolabs, NEB, USA) and 0.1 µl of Exonuclease I (0.5 u/µl; NEB, USA) and incubation at 37°C for 1 hour and 80°C for 20 min. Purified DNA fragments were sequenced using the Big Dye v3.1 Terminator Sequencing kit (PE Applied Biosystems, Foster City, CA, USA), visualizing results on an ABI 310 automated sequencer. For each sample, *wsp* sequences were obtained from both strands and compared between them and with the reference sequence of *w*Dil and *w*VulC.

### Electron microscopy

To date, the most reliable way to characterize autophagy is the visualization by TEM of the formation of double membrane cytosolic vesicles called autophagosomes which sequesters cytoplasm and delivers it to the lysosome for degradation and are considered as the hallmark of autophagy [30], [42]. To observe CNS cells and gonads by TEM, 6 animals from each host species (*i.e A.vulgare* and *P. d. dilatatus*) and from each treatment (injected by *w*VulC, injected by *w*Dil, or injected by an ovary suspension containing no *Wolbachia*) were individually sampled 30 and 60 days post-injection. Tissues were fixed (9% glutaraldehyde, 0.3 M sodium cacodylate, 3% NaCl, v/v/v) for 2 hours at 4°C. Tissues were washed (0.3 M sodium cacodylate, 3% NaCl, 0.8 M sucrose, v/v/v) for 2 hours at 4°C. Post-fixation was performed into 4% OsO_4_, 0.3 M sodium cacodylate, 5.5% NaCl for 45 min. Tissues were subsequently dehydrated through a graded series of acetone solutions, infiltrated and embedded in resin (Spurr, Polyscience Inc.). Thick sections (0.5 µm) were stained with 1% toluidin blue for light microscopy observation. Thin sections (90 nm) were contrasted by incubation in 1% uranyl acetate in 50% ethanol for 1 min, then stained with lead citrate [43]. Sections were observed using a transmission electron microscope (JEOL 100C).

### LC3B labeling antibody

The LC3B protein plays a critical role in autophagy. Normally, this protein resides in the cytosol, but following cleavage and lipidation with phosphatidylethanolamine, LC3B associates with the phagophore. This localization can be used as a general marker for autophagic membranes. To visualize the LC3B, we used the LC3B Antibody Kit for Autophagy (Invitrogen). As this kit was designed to work with cell coming from cell culture it was not possible to perform it on the full CNS. We thus only performed LC3B labeling on adipocytes from the nerve chord which form a loose tissue that can be more easily penetrated by antibodies. To perform this labeling, we first deposited individually part of fat tissue containing adipocytes collected in: 6 *A. vulgare* naturally infected by *w*VulC, 6 *A. vulgare* injected by *w*VulC, 6 *P. d. dilatatus* injected by *w*Dil and 6 *P. d. dilatatus* injected by *w*VulC (at 45 days post-injection) onto microscope slides harboring a 200 µL drop of 3.7% formaldehyde in PBS. The fat tissue samples were then incubated in fixative for 15 minutes at room temperature. The fixative was then removed and the adipocytes washed three times with PBS. After the last washing, 200 µL of 0.2% Triton X-100 in PBS were deposited onto the cells. The permeabilization buffer was then removed and 200 µL of the primary antibody (0.5 µg/mL in blocking BSA buffer) were deposited onto the cells and incubated for 1 hour at room temperature. The primary antibody was then removed and the samples washed again three times with PBS. The samples were then placed with 200 µL of an anti-rabbit secondary antibody (0.5 µg/mL in blocking BSA buffer) and incubated for 1 hour at room temperature. Samples were mounted in Citifluor (AFl antifading, Cititfluor, England). Detection was performed with a Carl Zeiss epifluorescent microscope (Axio Observer.Z1) with Apotome and AxioVision 4.8.1 software (Zeiss) equipped with a 63X objective (oil immersion).

### Statistical analysis

All statistical analyses were performed using R software (version 2.10.1, 2009-12-14). For all life-history traits, we first adjusted a model to our data which includes sex, dose (*i.e.* the number of *Wolbachia* injected), and treatments (control, *w*VulC or *w*Dil). We then re-adjusted the same model after having removed the control treatment from the data set, which allowed us to compare between the effects of *w*VulC and *w*Dil. As the experiments were performed on three independent groups of individuals, a randomized block effect has been taken into account in the models when possible. This last analysis showed that the inter-block variance was very low indicating that measures were repeatable between blocks. The gain of weight and mobility were compared between treatments at 60 days post-injection with a Gaussian linear mixed-effects model with randomized block effect fit by maximum likelihood (ML) (lme function in nlme package [44]). For comparison of the animals' location in the boxes between treatments through the whole experiment, a generalized linear model with binomial error and logit link function was performed. In these analyses, the data were collected from simultaneous observations of all the individuals from a same rearing box. The comparison was thus made between groups of 6 boxes and not between individuals, it was thus not necessary to add a random effect in this model. Survival curves obtained with the different treatments were compared using a general mixed-effects Cox model with randomized block effect (coxme fonction in kinship package). *Wolbachia* density in tissues of *A. vulgare*, *P. d. dilatatus* and naturally infected animals were compared with t-test because data distribution followed a normal distribution and variances of the samples were homogeneous.

## Supporting Information

Video S1 **Behavioral film showing the symptoms encountered in** ***P. d. dilatatus*** **after the injection of the** ***w*** **VulC strain.** Animals exhibited surfacing behavior, lack of mobility and several symptoms as seizures, leg tremors and paralysis.
(MP4)
